# Are interventional radiology techniques ideal for nonpenetrating splenic injury management: Robust statistical analysis of the Trauma Quality Program database

**DOI:** 10.1371/journal.pone.0315544

**Published:** 2024-12-31

**Authors:** Randeep S. Jawa, Amit Gupta, James Vosswinkel, Marc Shapiro, Wei Hou

**Affiliations:** 1 Department of Surgery, Stony Brook University Renaissance School of Medicine, Stony Brook, New York, United States of America; 2 Department of Radiology, Ohio State University, Columbus, Ohio, United States of America; 3 Department of Family Health and Preventive Medicine, Stony Brook University Renaissance School of Medicine, Stony Brook, New York, United States of America; The Fourth Affiliated Hospital Zhejiang University School of Medicine, CHINA

## Abstract

**Background:**

Splenic artery embolization (SAE) is increasingly favored for adult blunt splenic injury management. We compared SAE to other splenic injury management strategies using robust statistical techniques.

**Materials and methods:**

Univariate analyses of demographics and outcomes were performed for four patient groups: observation, SAE, splenic surgery, splenic surgery + SAE in the American College of Surgeons Trauma Quality Program (TQIP) database. To address nonlinear associations of ED vital signs with mortality, multivariable spline-based logistic regression models with interaction terms between hemodynamic status and management strategy and either splenic Abbreviated Injury Score (AIS) or Injury Severity Score (ISS), were generated.

**Results:**

In 44,187 splenic injury patients meeting study inclusion criteria, the most common management strategy was observation alone (77.9%). The observation group had median spleen AIS of 2, ISS 20, with 6.3% mortality; SAE (2.6%) had median spleen AIS3, ISS 24, with 6.6% mortality; splenic surgery (22.4%) AIS4, ISS 29, with 15.4% mortality; and splenic surgery + SAE (0.04%) AIS4, ISS 29, with 15.2% mortality. In multivariable models, SAE had lower predicted probability of mortality than surgery over most initial ED systolic blood pressures (SBPs). At all spleen AIS, SAE had lower predicted mortality than surgery. SAE had lower mortality than surgery except at very high ISS, where it was comparable. SAE had lower predicted mortality than observation management at spleen AIS≥3. In subgroup analysis of patients without severe multi-system injuries, predicted mortality did not differ by management strategy.

**Conclusions:**

SAE is associated with decreased mortality at spleen AIS 3–5. The benefits of SAE appear to be largely for spleen AIS 3–5 in the setting of severe (AIS≥3) multi-system injuries.

## Introduction

Observation alone following blunt splenic injury (BSI) was associated with substantial failure rates in the past, prompting new approaches, particularly the use of splenic artery embolization (SAE) for high grade splenic injuries [1–8]. However, SAE is not benign and has been associated with a variety of complications including those at the access site, such as hematoma, and at the spleen, such as abscess [6, 7, 9]. Although there is extensive literature on splenic injury management, further study is indicated for three reasons. First, in 2018, the American Association for the Surgery of Trauma (AAST) changed the splenic injury grading scale (ie Abbreviated Injury Score (AIS) which ranges from 1–5 for splenic injury), so that splenic artery pseudoaneurysms and arteriovenous fistulas would be categorized as spleen AIS 4 or 5 instead of spleen AIS 3 before the update [10]. However, the AIS coding manual used for recording splenic injury in national trauma databases is based on splenic injury scoring that relies on previous splenic injury definitions which do not consider these 2 vascular abnormalities, ie these injuries would receive a splenic injury grade of 3 [11]. Because of this discrepancy in scoring and coding, and therefore potentially altered management strategy, we examined data from 2013–2016 in a US/Canadian trauma database. Second, conventional multivariable logistic regression analyses of splenic injury management on hospitalization outcomes do not account for the nonlinear relationships between vital signs and outcomes nor do they account for the potential interaction between splenic injury management and splenic injury severity. In other words, splenic injury management and splenic injury severity are considered as independent variables, when these variables may be dependent on each other. Third, other injuries in the abdomen and/or other body regions may also influence splenic injury management, and consequently the overall injury burden and splenic injury management may also be dependent on each other in multivariable logistic regression analyses. We therefore determined how splenic injury grade, injuries in other body regions, and hemodynamic status interplay in splenic injury management and outcomes using robust statistical modeling techniques, i.e. is there an optimal management group that is suited for SAE?

## Methods

Data from 2013 to 2016 were derived from the American College of Surgeons Trauma Quality Program (ACS TQP), which contains information from submitting facilities in US and Canada, was analyzed for adults (age≥18 years) with nonpenetrating splenic injury with a spleen abbreviated injury score (AIS) ≥2. The ACS TQP participant use files (PUF) contain a combination of data from Trauma Quality Improvement Project (TQIP) and the National Trauma Data Bank (NTDB) research dataset. Congruent with the US Department of Health and Human Services 45 CFR 46 Institutional Review Board review is not required of data available for public use and not individually identifiable and therefore would not involve human subjects. There is no private information in this dataset and no attempt to link with any other dataset. Hence, with regards to ethics approval, this research is classified as quality improvement. Concomitantly, as the dataset is fully deidentified, there would be a waiver of consent by participants. There is no way for us to obtain participant consent as we do not know the participants in this public use dataset. This cross-sectional study is consistent with Strobe guidelines.

Management strategies were divided into four categories: observation (ie observation alone with no splenic surgery nor SAE) (Group 1); SAE (Group 2, as identified by site of embolization in the dedicated TQP embolization site table); splenic surgery (splenorrhaphy and/or splenectomy as identified by ICD-9 or ICD-10 codes) (Group 3); and a combination of splenic surgery and SAE (Group 4). The observational group was separated from the SAE group to discern the benefits of each approach.

Data cleaning, validation, and statistical analyses of variables of interest were performed by team members with training in analytics/biostatistics. As this is an administrative database, individual chart review was not possible. Of 70,560 patients identified, we excluded deaths in the Emergency Department (ED, n = 1809) and those with missing data for ED systolic blood pressure (SBP, n = 471), ED pulse (n = 1038), spleen AIS (n = 295), and Injury Severity Score (ISS, n = 1507), yielding 65,440 patients. Per the NTDS, recorded ED vitals should be the index vitals. Multiple imputation of the missing values was considered but decided against because of the potential for confounding by generation of synthetic data. To assure data quality, only the subset of patients meeting TQIP inclusion criteria (n = 41,187), which are identified as such in the PUF, were examined. Consistent with the AIS methodology for determining injury severity in each of the 6 body regions (head/neck, face, chest, abdomen including pelvic contents, extremities including the bony pelvis, and external), the highest AIS severity score was selected and assigned for the region. If there was no AIS value for a body region, it was given an AIS score of 0, as this would mean that the patient did not have an injury in that region, by convention. With regards to Injury Severity Score (ISS), a global measure of injury severity determined by summing the maximal AIS in the 3 most severely injured body regions, the value was derived from the maximal AIS in three of the 6 most severely injured body regions.

The following National Trauma Data Standard (NTDS) defined comorbidities were examined: bleeding disorder, cerebrovascular accident/residual neurologic deficit, diabetes mellitus, history of myocardial infarction, cirrhosis, and functional dependence [12]. The following NTDS complications were included: unplanned intubation, myocardial infarction, deep venous thrombosis, pulmonary embolism, cardiac arrest with resuscitative efforts by healthcare provider, deep surgical site infection, organ/space surgical site infection, pneumonia, ventilator associated pneumonia, superficial surgical site infection, urinary tract infection, catheter related blood stream infection, unplanned return to the operating room, unplanned intensive care unit (ICU) admission, severe sepsis, acute kidney injury, acute lung injury/acute respiratory distress syndrome. There is some variation of these definitions over time. A binary variable was created to account for the presence of any complication.

Data was analyzed using SAS (North Carolina, USA) and R (Vienna, Austria). Univariate (chi square and Kruskal Wallis) statistics were calculated. Linear, independent, correlations were examined. Multivariate generalized additive logistic regression models (GAM) were also generated. Variables included in the logistic regression models were index emergency department (ED) pulse, index ED SBP, age, blood transfusion within 4 hours, plasma transfusion within 4 hours, platelet transfusion within 4 hours, management group, comorbidities, and ISS. Other than management strategy, the predictors used in these models are pre-existing factors or those that occur early after presentation. The addition of hospitalization characteristics such as length of stay and complications would likely have increased the models’ predictive accuracy, however, would be less useful proactively when determining care options. Because of collinearity between ISS and spleen AIS, i.e. as spleen AIS increases the ISS increases, separate multivariate models that excluded ISS but included spleen injury severity (i.e. spleen AIS) and AIS in non-abdominal regions were generated. In these Generalized Additive Models (GAM), splines were used for index ED pulse and index ED SBP, because these 2 variables demonstrated a nonlinear association with mortality, i.e. mortality increases at either extreme of index pulse rate or index SBP. GAM models enable demonstration of these findings because they are piecewise polynomials stitched together.

Because interpretation of odds ratios with interaction terms in GAM models is more complex, they were graphically represented via estimated marginal means (or medians) interaction plots which have 95% confidence intervals. Further, unlike simple logistic regression tables which display comparisons with only one specific group, estimated marginal mean interaction plots compared medians at each splenic AIS or ISS and management strategy. Interactions were also generated between management group and index ED SBP.

## Results

### Splenic injury management distribution

Of the 41,187 patients with complete information of the key variables, the management distribution was 30731 (74.6%) group 1 (observation), 1089 (2.6%) group 2 (SAE), 9209 (22.4%) group 3 (splenic surgery: 8457 splenectomy, 684 splenorrhaphy, 68 both), and 158 (0.04%) group 4 (splenic surgery + SAE) (Table 1). Of the 68 patients that had both splenorrhaphy and splenectomy, in 28 patients, the splenorrhaphy preceded splenectomy by hours to days. In another 28 cases the two were noted to have occurred at the same time, with the possibility that perhaps splenorrhaphy was attempted in the OR but aborted and converted to splenectomy, or one of the 2 codes is erroneous. In 9 cases, the splenectomy preceded the splenorrhaphy, hence erroneous coding, with possibilities including that splenorrhaphy occurred first followed by splenectomy. In 2 cases, the time for splenorrhaphy is recorded but not that for splenectomy. Amongst group 3 patients, 73.9% underwent splenic surgery within 4 hours; 120 patients underwent splenic surgery after 7 days. Observational management accounted for the majority of patients at level I-III ACS verified trauma centers and at those where ACS level was not available (Table 1). The rate of SAE was low (0%-3.1%) at all ACS Adult Trauma Center Verification Levels; SAE or SAE + splenic surgery was never performed at Level III centers.

**Table 1:** Summary statistics by management group. Percentages are based on the values on the overall number of patients in each column, unless otherwise indicated. P-values indicate differences amongst groups. ISS: injury severity score; AIS: abbreviated injury score; SAE: splenic artery embolization.

	observation	SAE	splenic surgery	splenic surgery + SAE	P value
Number	30731	1089	9209	158	-
Age	38 [25–55]	44 [29–58]	41 [27–55]	42.5 [27.25–59]	<0.0001
ISS	20 [14–29]	24 [17–34]	29 [20–38]	29 [21–43]	<0.0001
Spleen AIS	2 [2–3]	3 [3–4]	4 [3–4]	4 [3–5]	<0.0001
Abdomen AIS	3 [2–3]	3 [3–4]	4 [3–5]	4 [3–5]	<0.0001
Chest AIS	3 [1–3]	3 [0–3]	3 [1–3]	3 [1–4]	<0.0001
Head/neck AIS	0 [0–3]	0 [0–3]	0 [0–3]	0 [0–3]	0.84
Extremity AIS	2 [0–2]	2 [0–2]	2 [0–3]	2 [0–2.75]	0.53
External AIS	0 [0–0]	0 [0–0]	0 [0–0]	0 [0–0]	0.77
Index ED PULSE	94 [80–110]	98 [82–116]	102 [84–121]	104.5 [86.25–121]	<0.0001
Index ED SBP	126 [110–142]	116 [100–135]	112 [92–132]	110 [91–132]	<0.0001
Blood transfusion 4 hours (%)	13.9%	76.0%	52.4%	84.2%	<0.0001
Plasma transfusion 4 hours (%)	7.91%	28.1%	36.7%	48.7%	<0.0001
Platelet transfusion 4 hours (%)	4.7%	13.4%	24.7%	31.0%	<0.0001
Spleen AIS2 (%)	58.2%	16.7%	19.9%	15.2%	-
Spleen AIS3 (%)	29.1%	40.7%	26.8%	25.9%	-
Spleen AIS4 (%)	10.1%	32.7%	30.4%	28.5%	-
Spleen AIS5 (%)	2.6%	9.9%	22.9%	30.4%	-
Complications (%)	17.8%	22.0%	34.0%	43.0%	<0.0001
Spleen AIS2 (%) (complications/total)	20.7% (3709/17888)	31.3% (57/182)	43.0% (791/1837)	58.3% (14/24)	<0.0001
Spleen AIS3 (%) (complications/total)	13.1% (1175/8932)	17.8% (79/443)	33.2% (820/2467)	31.7% (13/41)	<0.0001
Spleen AIS4 (%) (complications/total)	13.6% (423/3107)	21.1% (75/356)	20.7% (861/2799)	48.9% (22/45)	<0.0001
Spleen AIS5 (%) (complications/total)	22.0% (177/804)	26.8% (29/108)	31.3% (660/2106)	39.6% (19/48)	<0.0001
Mortality (%)	6.3%	6.6%	15.4%	15.2%	<0.0001
Spleen AIS2 (%) (died/total)	7.5% (1344/17888)	11.5% (21/182)	23.0% (423/1837)	29.2% (7/24)	<0.0001
Spleen AIS3 (%) (died/total)	3.9% (347/8932)	7.0% (31/443)	14.3% (354/2467)	9.8% (4/41)	<0.0001
Spleen AIS4 (%) (died/total)	4.0% (125/3107)	3.4% (12/356)	11.6% (324/2799)	11.1% (5/45)	<0.0001
Spleen AIS5 (%) (died/total)	14.7% (118/804)	7.4% (8/108)	15.1% (319/2106)	16.7% (8/48)	0.84
ACS Level I	74.5% (14491)	2.6% (501)	22.6% (4386)	0.4% (82)	-
ACS Level II	75.3% (5539)	3.1% (228)	21.3% (1572)	0.3% (24)	-
ACS Level III	85.3% (262)	0% (0)	14.6% (45)	0% (0)	-
ACS Level N/A	74.3% (10035)	2.5% (343)	22.8% (3074)	0.4% (50)	-

The transfusion of blood/plasma/and platelets within 4 hours of ED presentation most often occurred in the SAE + splenic surgery group. Of note, however, while SAE had higher rates of blood transfusion than the surgical group; the latter had higher rates of plasma and platelet transfusion. The observation group had the lowest rates of all transfusions.

### Splenic injury distribution

The median spleen AIS ranged from 2 in the observation group, 3 in the SAE group, to 4 in both surgical groups (ie surgery ± SAE). Further, the ISS progressively increased from observation (22) to SAE (26) to surgical groups (ie surgery ± SAE) (29). With increasing spleen AIS, the rate of observation management decreased (Table 1, S1 Fig). Surgical groups (with or without SAE) had a greater preponderance of higher spleen severity scores in association with very high ISS (51+) scores, whereas the observation group had lower splenic injury severity scores even at very high ISS (51+) scores (S1 Fig).

### Outcomes

The overall in-hospital complication rate increased from 17.8% in the observation group to 22.0% in SAE, 34.0% in surgery, and 43.0% in surgery + SAE group (Table 1). Upon further stratification by splenic injury grade, the surgery groups with or without SAE had the highest complication rates, followed by SAE alone, which had higher complication rates than observational management at all spleen AIS. The overall in-hospital mortality rate was 8.4%, ranging from 6.3% in the observation group to 6.6% in SAE, 15.4% in the surgery group, and 15.2% in surgery + SAE. Stratified by splenic injury grade, splenic surgery groups with or without SAE had the highest rate of mortality at spleen AIS 2–4. Mortality rates with SAE were higher than observation at AIS2 and AIS3, but lower at AIS4 and AIS5. A linear correlation matrix was also constructed (S2 Fig). Notably, spleen injury severity had minimal correlation with complications (0.01) or death (0.02). It demonstrated that overall injury severity (as measured by the ISS) had the strongest correlation with complications (r = 0.29) and death (r = 0.31).

**Fig 1:**
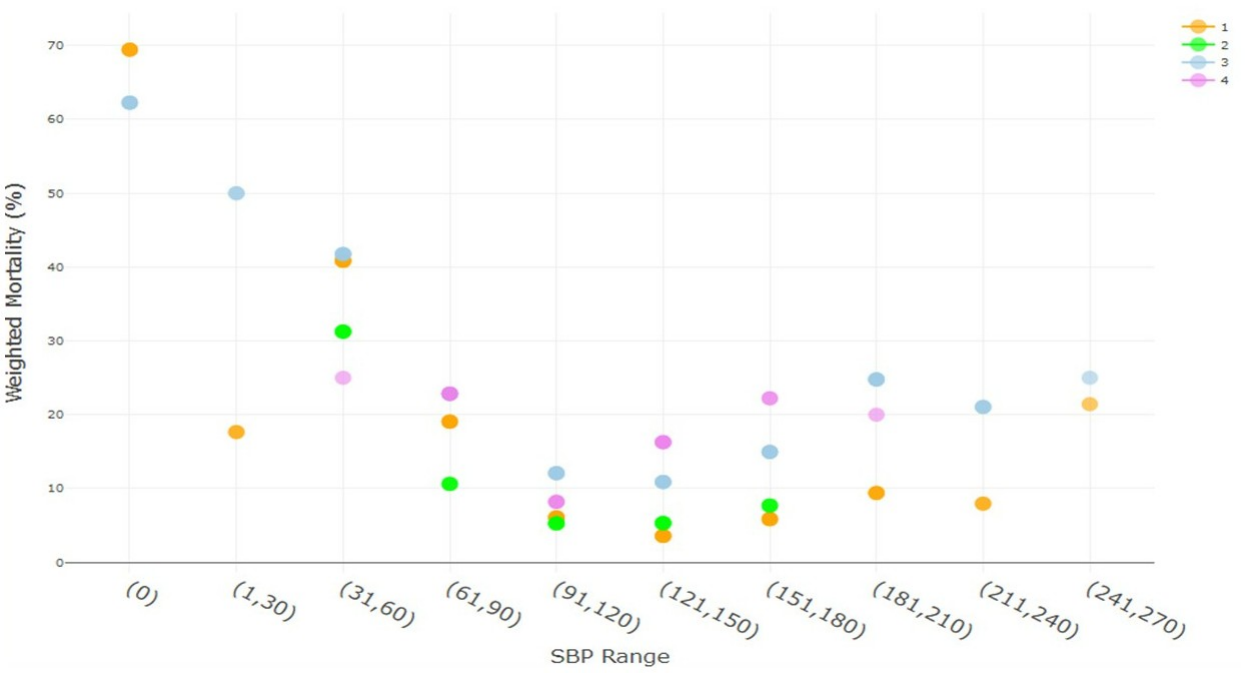
Raw data demonstrating a bimodal association of initial ED SBP range with weighted mortality for all patients (n = = 41187) by management group, whereby higher mortality is noted at very low and very high initial ED SBP range. The raw data demonstrates the need to use splines to account for the nonlinear relationship between initial ED SBP and mortality in multivariate regression. The mortality percentage at each initial ED SBP range is the weighted mean mortality, whereby the group mortality is weighted by the number of patients at each level of splenic severity. Group 1 (orange)—observation, group 2 (green)—SAE, group 3 (blue)–splenic surgery, group 4 (purple)–splenic surgery + SAE.

**Fig 2:**
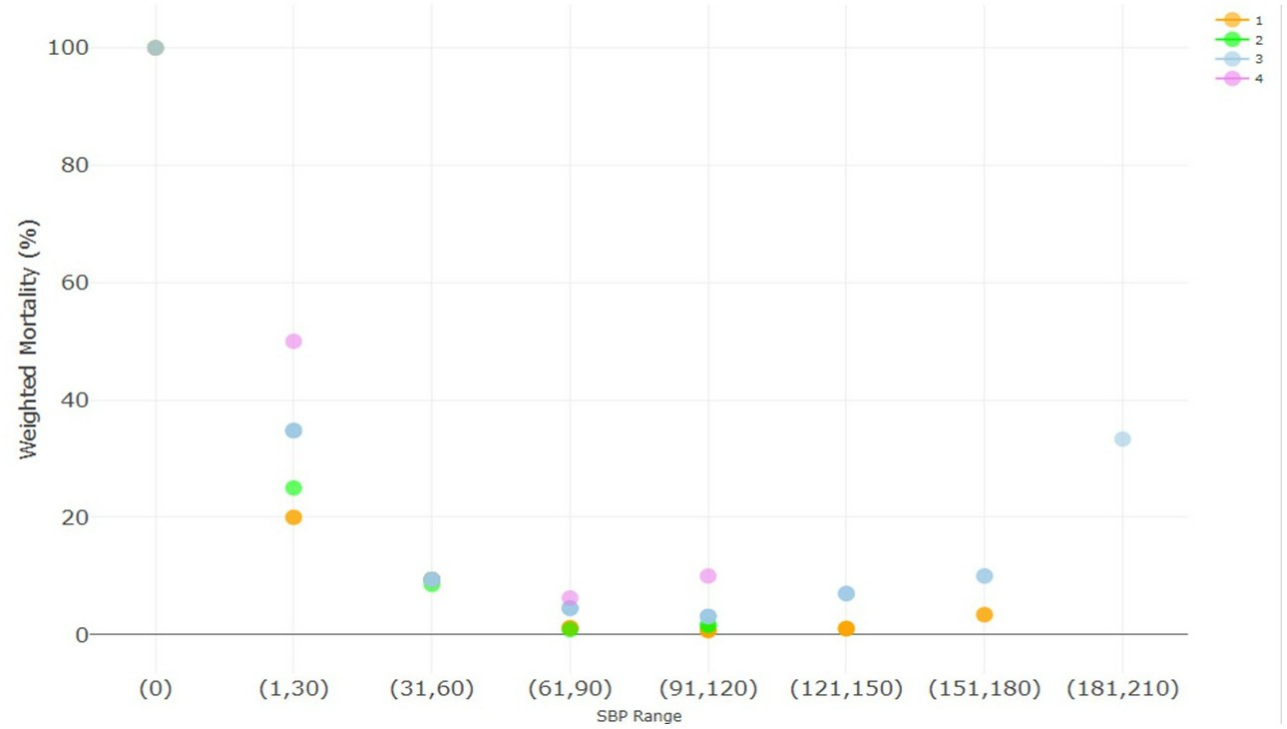
Raw data demonstrating a bimodal association of initial ED SBP range with mortality for patients (n = 8703) without severe injuries (AIS<3) in non-abdominal body regions by management group (n = 8703), whereby higher mortality is noted at very low and very high initial ED SBP range. The raw data demonstrates the need to use splines to account for the nonlinear relationship between initial ED SBP and mortality in multivariate regression. The mortality percentage at each initial ED SBP range is the weighted mean mortality, whereby the group mortality is weighted by the number of patients at each level of splenic severity. Group 1 (orange)—observation, group 2 (green)—SAE, group 3 (blue)–splenic surgery, group 4 (purple)–splenic surgery + SAE.

Mortality, weighted by the number of patients at each splenic injury severity and management group, at the ranges of index ED SBPs, were examined. As seen in Fig 1, there is a bimodal mortality curve for all management groups, whereby mortality is highest at very low SBP (≤60 mmHg), then decreases, and then increases again at high SBP (≥151 mmHg); however, not all management groups are represented at either extreme of SBP. Other than at an initial ED SBP of 0, the observational management had less mortality than surgery throughout the range of SBP. SAE has lower mortality than observational management and surgery at low SBP. When considering SBP in the 8703 patients with splenic injury and non-severe injuries (ie AIS≤2) in the 5 non-abdominal body regions, the bimodal mortality curve was again noted (Fig 2). Furthermore, in both cases, splenic surgery (with or without SAE) generally had a higher weighted mortality than observation or SAE across the range of SBP.

### Multivariate analyses of predictors for inpatient mortality

Multivariable cubic spline generalized additive logistic regression models were generated to determine the effects of management strategy, initial hemodynamics, comorbidities, injuries and other covariates on in-patient mortality. One model was based on ISS as the overall measure of injuries (Table 2). This model demonstrated that ISS was significantly directly associated with mortality (OR 1.09). Without considering the interaction with ISS, both SAE (OR 1.06) and splenic surgery (OR 3.1) were significantly associated with increased probability of death. However, when considering the interaction of management group with ISS, SAE (OR 0.98), splenic surgery (OR 0.97), and surgery + SAE (0.96) were all significantly inversely associated with death. Among the comorbidities examined, the presence of cirrhosis had the highest odds ratio (4.20) for death. Transfusion of blood, plasma, or platelets within 4 hours of hospitalization were also significantly associated with mortality. In these regression models, adult trauma center ACS verification level was not included because of the very few (n = 307) splenic injury patients, especially those with higher grades (36 grade 4 and 25 grade 5 patients), treated at level III centers and absence of SAE at these centers.

**Table 2:** Generalized Additive regression Models (GAM) with cubic regression splines for ED SBP and ED Pulse, using ISS or spleen injury severity to predict in-hospital mortality. Note that the presence of the interaction terms between ISS and management group (Model 1) or splenic injury severity and management group (Model 2).

	Model 1: Injury	Severity Score	Model 2: Spleen	Injury Severity
	OR [95% CI]	P-value	OR [95% CI]	P-value
Observation	Group Reference	Group Reference	Group Reference	Group Reference
SAE	1.06 [0.54–2.08]	0.9	0.95 [0.92–0.99]	0.011
Surgery	2.51 [2.00–3.16]	<0.001	1.07 [1.06–1.09]	<0.001
Surgery + SAE	2.18 [0.57–8.26]	0.3	1.09 [0.98–1.20]	0.11
Age	1.03 [1.03–1.03]	<0.001	1.00 [1.00–1.00]	<0.001
Blood Transfusion in 4hrs	1.58 [1.39–1.80]	<0.001	1.03 [1.03–1.04]	<0.001
Plasma Transfusion in 4hrs	1.55 [1.34–1.79]	<0.001	1.07 [1.06–1.08]	<0.001
Platelet Transfusion in 4 hrs	1.62 [1.43–1.83]	<0.001	1.12 [1.10–1.13]	<0.001
Bleeding Disorder	1.47 [1.24–1.76]	<0.001	1.03 [1.02–1.04]	<0.001
Diabetes Mellitus	0.99 [0.86–1.14]	0.9	0.99 [0.98–1.00]	0.081
Functional Dependence	1.06 [0.74–1.53]	0.7	1.00 [0.97–1.02]	0.8
Cirrhosis	4.05 [3.23–5.07]	<0.001	1.16 [1.13–1.18]	<0.001
MI History	1.52 [1.05–2.20]	0.028	1.03 [1.00–1.06]	0.053
ISS	1.08 [1.08–1.08]	<0.001	n/a	n/a
ISS*SAE	0.98 [0.96–1.0]	0.014	n/a	n/a
ISS*Surgery	0.97 [0.97–0.98]	<0.001	n/a	n/a
ISS*(Surgery+SAE)	0.96 [0.93–1.00]	0.038	n/a	n/a
Head/neck AIS	n/a	n/a	1.04 [1.03–1.04]	<0.001
Face AIS	n/a	n/a	1.00 [1.00–1.00]	0.4
Chest AIS	n/a	n/a	1.01 [1.00–1.01]	<0.001
Extremity AIS	n/a	n/a	1.00 [0.99–1.00]	<0.001
External AIS	n/a	n/a	1.01 [1.01–1.02]	<0.001
Spleen AIS2	n/a	n/a	AIS Reference	AIS Reference
Spleen AIS3	n/a	n/a	1.00 [1.00–1.01]	0.7
Spleen AIS4	n/a	n/a	1.00 [0.99–1.01]	0.8
Spleen AIS5	n/a	n/a	1.06 [1.05–1.07]	<0.001
AIS2*Observation	n/a	n/a	Interaction term Reference	Interaction term Reference
AIS3*SAE	n/a	n/a	1.00 [0.96–1.04]	>0.9
AIS3*Surgery	n/a	n/a	0.95 [0.93–0.96]	<0.001
AIS3*(Surgery+SAE)	n/a	n/a	0.87 [0.76–0.98]	0.024
AIS4*SAE	n/a	n/a	0.98 [0.94–1.03]	0.5
AIS4*Surgery	n/a	n/a	0.92 [0.90–0.94]	<0.001
AIS4*(Surgery+SAE)	n/a	n/a	0.90 [0.80–1.02]	0.11
AIS5*SAE	n/a	n/a	0.95 [0.89–1.01]	0.094
AIS5*Surgery	n/a	n/a	0.88 [0.86–0.90]	<0.001
AIS5*(Surgery+SAE)	n/a	n/a	0.84 [0.74–0.95]	0.005
spline (index ED SBP)	-	<0.001	-	<0.001
spline (index ED Pulse)	-	<0.001	-	<0.001

As there were 11527 transfer patients, we repeated this analysis with transfer status as a variable, excluding the 8 patients where transfer status was not known, and noted that transfer status was significantly inversely associated with mortality (OR 0.76 [95% CI 0.68–0.84], p<0.001). We also performed an additional GAM regression and excluded the 11527 transfer patients and 8 patients where transfer status was not known. The overall findings were similar, with the exception of the interaction of SAE with ISS, while the values remained identical (OR 0.98 [95% CI 0.96–1.00]), the p-value increased from 0.014 to 0.10 with the reduced SAE group size (n = 769).

We also repeated the GAM analysis using mechanism of nonpenetrating injury as a predictor, where the most common mechanism of injury was motor vehicle/motorcycle accidents (n = 21047), followed by falls (n = 4284), other transport accidents (n = 2078), pedal cycle other/pedestrian other (n = 491), struck by/other (n = 1177). However, 11996 patients did not have a mechanism specified and were excluded from the regression. In this analysis, as compared to falls, only motor vehicle/motor cycle accidents had a higher OR (1.24 [95% CI 1.06–1.45], p = 0.006) and struck by/other had lower OR (0.42 [95% CI 0.25–0.70], p<0.001).

The second multivariate GAM regression model examined management group by spleen AIS with maximal AIS scores in the non-abdominal body regions (Table 2). Spleen AIS 5 (the most severely injured spleen) had an 1.06 odds ratio for death as compared to spleen AIS2. Among the comorbidities, cirrhosis again had the highest odds ratio (1.16) of death. Transfusion of blood, plasma, or platelets within 4 hours of hospitalization were again significantly associated with mortality.

**Fig 3:**
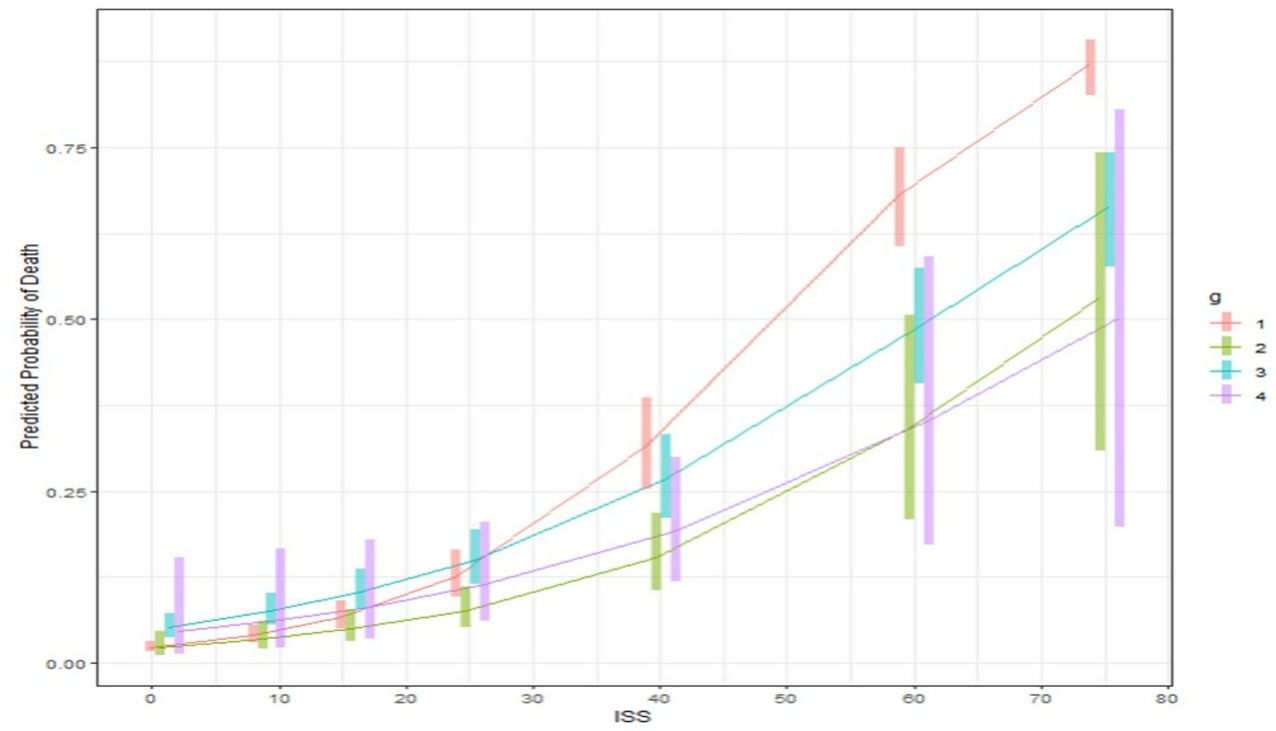
Interaction plot with 95% confidence intervals generated from GAM regression model 1 in Table 2 examining ISS and management group interactions. Group 1: observation, Group 2: SAE, Group 3: splenic surgery, Group 4: splenic surgery + SAE. The intersecting management strategy (group) lines confirm the presence of an interaction between these 2 variables and demonstrate the need for more complex logistic regression models that consider these interactions. Results are averaged over the levels of the other variables in the models. At high ISS (>51), observational management has the highest mortality.

**Fig 4:**
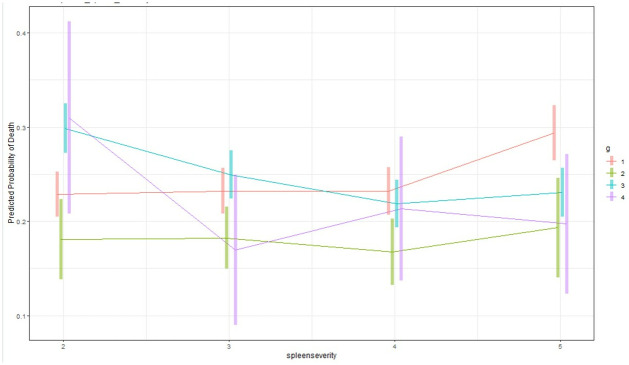
Interaction plot with 95% confidence intervals generated from the GAM model 2 in Table 2 examining spleen AIS and management group interactions. Group 1: observation, Group 2: SAE, Group 3: splenic surgery, Group 4: splenic surgery + SAE. The intersecting management strategy (group) lines confirm the presence of an interaction between these 2 variables and demonstrate the need for more complex logistic regression models that consider these interactions. Results are averaged over the levels of the other variables in the models. At spleen severity 2: observation and SAE have lower mortality than surgery (p<0.0001, p<0.0001). At spleen severity 3,4,5: SAE has lower mortality than observation (p = 0.005, p = 0.0005, p = 0.008) and surgery (p<0.0001, p<0.0001, p = 0.027).

Given the interactions of ISS with management group in the GAM model, we constructed an interaction plot that graphically depicted outcomes by management group at each ISS of interest (Fig 3). The presence of lines that intersect confirmed the interaction between spleen management strategy and ISS. The predicted probability of death dramatically increases for all groups with increasing ISS. Among the management strategies, SAE has the lowest predicted probability of in-hospital death once ISS ≥ 16. As assessed by nonoverlapping 95% confidence intervals, splenic surgery has higher mortality as compared to observational management at lower ISS scores but then becomes lower than observational management at very high ISS (51+). As expected, surgery + SAE group had very wide confidence intervals, given low numbers of patients. The interactions between management group, spleen AIS, and mortality are also demonstrated in the interaction plot generated from the second GAM regression model (Fig 4). SAE had lower mortality than surgery at all spleen severities, but lower mortality than observational management only at spleen AIS 3–5.

**Fig 5:**
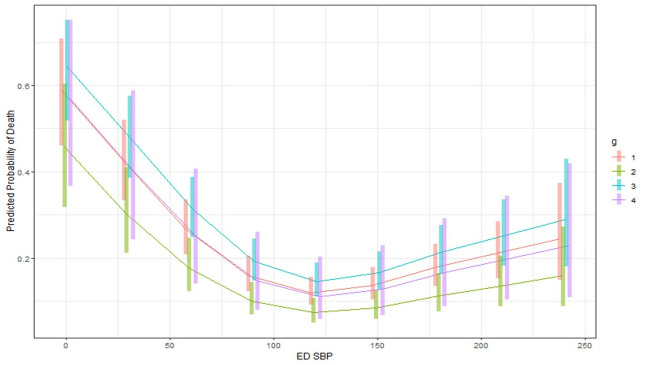
Interaction plot with 95% confidence intervals generated from the GAM regression model 1 in Table 2 examining initial ED SBP and management group interactions. Group 1: observation, Group 2: SAE, Group 3: splenic surgery, Group 4: splenic surgery + SAE. Results are averaged over the levels of the other variables in the models. Based on confidence intervals, SAE has lower mortality than surgery over a large range of ED SBP.

Next, an interaction plot of index ED SBP and management group was constructed. It paralleled the raw data and demonstrated a bimodal mortality curve and also demonstrated that surgery had similar mortality or higher mortality than SAE, with more pronounced differences at normotension (Fig 5).

**Fig 6:**
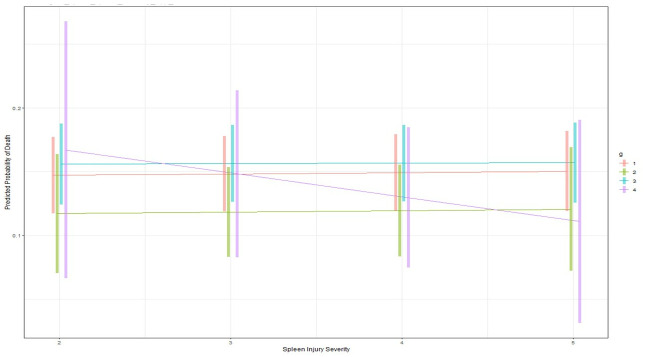
Interaction plots for probability of in-hospital death by management group and spleen severity in patients (n = 8073) with AIS≤ = 2 in head/neck, face, chest, extremity, and external regions. The presence of interactions between management group and spleen severity is demonstrated as the lines (i.e. management groups) intersect and thereby demonstrate that the interaction needs to be examined in multivariate regression models. All covariates in the original Table 2 were included. Group 1: observation (n = 6118, death = 86 (1.4%)), Group 2: SAE (n = 308, death = 7 (2.3%)), Group 3: surgery (n = 2235, death = 131 (5.9%)), Group 4: surgery+ SAE (n = 42, death = 3 (7.1%)). The only significant differences in outcomes were at spleen AIS = 2, where the very few patients in the surgery + SAE group had higher predicted probability of death than observation (p = 0.04), SAE (p = 0.007) and surgery groups (p = 0.04).

**Fig 7:**
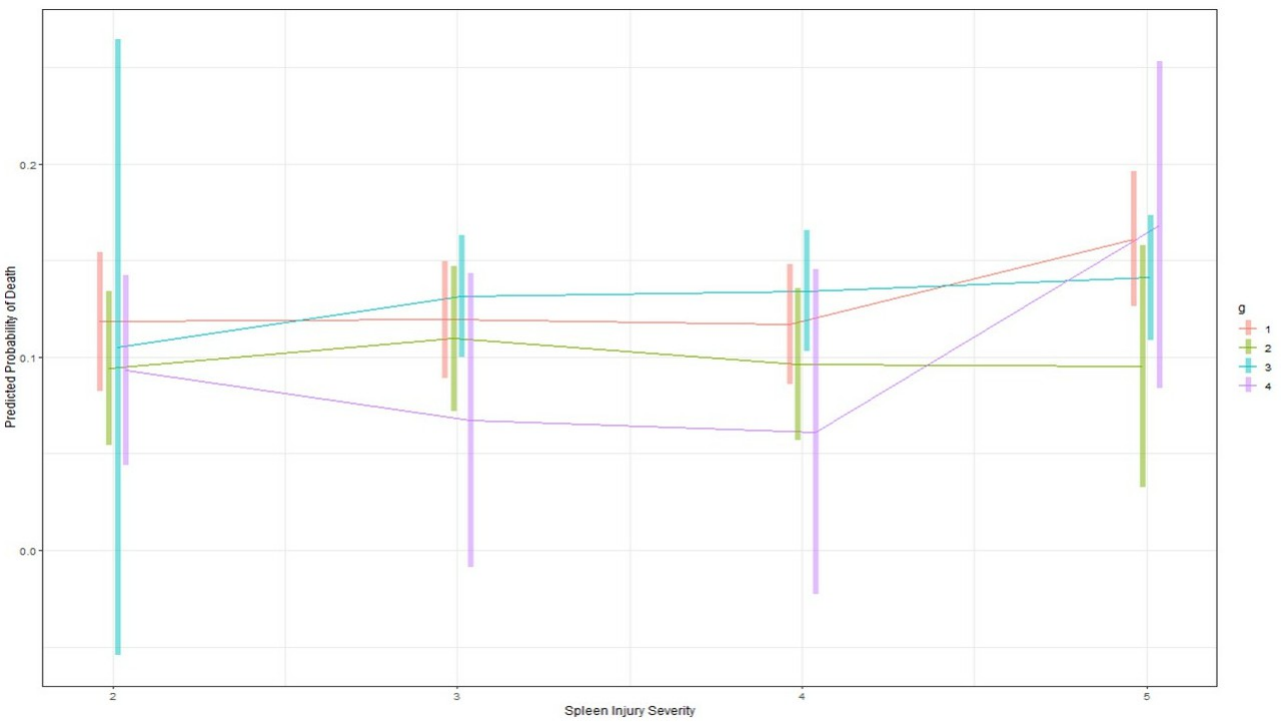
Interaction plots for probability of in-hospital death by management group and spleen severity in patients (n = 7210) with AIS≤ = 2 in head/neck, face, chest, extremity, and external regions and spleen injury AIS was the same as abdomen AIS. The presence of interactions between management group and spleen severity is demonstrated as the lines (i.e. management groups) intersect and thereby demonstrate that the interaction needs to be examined in multivariate regression models. All covariates in the original Table 2 were included. Group 1: observation (n = 4912, death = 47 (1.0%)), Group 2: SAE (n = 274, death = 7 (2.5%)), Group 3: surgery (n = 1872, death = 106 (5.4%)), Group 4: surgery+ SAE (n = 37, death = 2 (5.1%)).

Finally, given the role that major injuries in other body regions factor in mortality, the subset of patients (n = 8073) who only had serious injuries in the abdomen, ie AIS≤2 in head/neck, face, chest, extremity, and external body regions, were evaluated. This yielded 6118 (75.8%) observation, 308 (3.8%) SAE, 2235 (27.7%) surgery, and 42 (0.5%) splenic surgery + SAE patients. As seen in the interaction plot with spleen AIS using the same variables as in Table 2, at all spleen AIS, none of the management groups had significantly different predicted probabilities of death, except for the very few group 4 patients at spleen AIS2 (Fig 6). To minimize the potential effects of splenic injury severity as not being the highest severity abdominal injury and hence potentially influencing management, a subset of the above patients where splenic injury AIS was the same as abdomen AIS were examined (n = 7210) (Fig 7). Once again, neither surgery nor SAE provided predicted mortality benefit, as compared to observational management, for all grades of splenic injury.

## Discussion

In summary, the robust statistical analyses, that the authors have not seen previously used in splenic injury management studies, determined the following: (1) the majority of patients received observational management; (2) use of interaction terms between splenic injury management strategy and splenic injury severity or overall injury severity, and index ED SBP demonstrated SAE as the preferred management strategy, with regards to in-hospital mortality, for spleen AIS 3–5 when patients have more severe injuries (AIS≥3) outside of the abdomen. SAE is associated with increased complications without mortality benefit over observational management at spleen AIS 2; (3) Meanwhile, surgery is associated with higher predicted mortality than SAE in all splenic severities. In the absence of severe multi-system injuries, surgery failed to demonstrate a mortality benefit over non-operative management. Further, surgery had a higher raw mortality rate than observation and SAE regardless of hemodynamic status, other than index ED SBP of 0 mmHg. Given the few patients with surgery + SAE, and consequently wide confidence intervals, this discussion focuses on observational management, SAE, and surgery alone.

Of the 41187 patients with nonpenetrating splenic injury, 74.6% were managed with observation alone. As compared to a previous TQIP study, this larger percent of observation alone patients is in large part from the inclusion of grade II and III blunt splenic injury [3]. Of the 12746 patients with spleen AIS 4 or 5 therein, 44% underwent observational management without SAE, 4.1% underwent SAE, and 51.1% had splenic surgery. Moreover, 76% of patients with spleen AIS 3 were treated non-operatively without angiography, albeit the Eastern Association for the Surgery of Trauma guidelines advocate angiography consideration in AIS = 3 blunt splenic injury and a 2013 meta-analysis finding AIS = 3 blunt splenic injury to be a predictor of nonoperative management failure [13, 14].

This data demonstrate the general superiority of SAE at spleen AIS3/4/5, with regards to mortality. The lack of mortality benefit, as compared to observational management, at lower grade splenic injury is not unexpected and is consistent with current management trends. Furthermore, in the absence of severe multi-system injuries, SAE also does not afford a mortality benefit over observational management. Additionally, SAE did not obviate the need for blood/blood product transfusion and the risks thereof, as over 76% of SAE patients received blood transfusion within 4 hours of ED presentation. However, given the 4-hour time frame, these transfusions were more likely prior to or during SAE as opposed to post-procedure. Perhaps surprisingly, the univariate data demonstrated that SAE affords lower mortality than splenic surgery at all spleen AIS. This favorable outcome as compared to splenic surgery persists throughout the range of ISS in multivariate analyses. These benefits, however, become inapparent when patients with severe injuries in other body groups are excluded, ie the mortality benefit over observation or surgical management of splenic injuries is secondary to the co-existence of severe injuries in other body regions.

Notably, the rate of complications (surgical site infections and non-surgical site) however was higher with SAE (21%) than observation management (14.3%) throughout the spleen AIS range. This is consistent with findings of a recent meta-analysis, although the complication rates were lower than those reported therein, i.e. SAE 38.1% and nonoperative management 18.6% [7]. The meta-analysis also recommended against routine SAE in patients with spleen AIS 1–3 injuries. Not only do our results not identify a mortality benefit for SAE at spleen AIS 2, but rather demonstrate harm, with increased complication rates.

Given the increased rate of nonoperative management and angiography reported over time, the rate of SAE was somewhat lower than expected [9, 15–17]. However, the numbers are consistent with findings from a recent study examining the TQIP database from 2013 to 2019 with spleen AIS 3–5 [16]. This paper expands on that TQIP study as after excluding patients with serious injuries in other body regions, no mortality benefit for splenic surgery was present. To partially address the possibility that laparotomy may have been performed to address other abdominal injuries and hence splenectomy was incident to other abdominal injuries, only patients with no major non abdominal injuries (i.e. AIS≤2) and where the spleen AIS was the same as abdomen AIS were examined. Once again, management strategy did not influence predicted mortality at all splenic grades. Hence, at spleen AIS 3/4/5 SAE should be considered primarily in the setting of severe injuries in other body regions. These findings add to the findings of a consensus document recently published by the World Society for Emergency Surgery which conditionally recommended splenic angiography as the first line treatment for hemodynamically stable patients with arterial blush regardless of spleen AIS and also recommended SAE for all spleen AIS 4/5 injuries [18].

With regards to splenic surgery, various time frames after ED presentation have been previously utilized as metrics, eg 4 hours for failure of initially planned nonsurgical management in the literature [3]. To this end, the majority (73.9%) of splenic surgery was performed within 4 hours. The few (120) cases performed after 7 days is important in that captures the risk of delayed splenic rupture. Recognizing that patients managed with splenic surgery were older, had lower index ED SBP, higher index ED pulse, and higher ISS, multivariate GAM cubic spline based logistic regression analyses were performed to determine the influence of splenic injury management on mortality. Splenic surgery (i.e. splenorrhaphy and splenectomy) was not associated with reduced mortality as compared to observational management, at all splenic AIS. These findings were corroborated in subsequent GAM regression analyses where all patients with severe (AIS≥3) injuries in body regions other than the abdomen were excluded. Arguably, this finding should be expected, because if splenic injury severity is minimally associated with mortality, then the rationale for splenic surgery (except perhaps in spleen AIS5) becomes unclear. Stratified by splenic injury grade, splenic surgery also had the highest rate of complications on univariate analyses. Hence, splenic surgery did not demonstrate mortality reduction vs observational management and also had increased complication rates. These findings should not be surprising given the lack of linear correlation between splenic injury severity and mortality or complications.

Mortality herein was significantly associated with increasing age (OR 1.03), as seen in some other studies [19, 20]. Mortality was also associated with transfusion of blood and blood products, as well as a variety of comorbidities, principal among them being cirrhosis. Cirrhosis has been previously identified as a risk factor for failure of nonoperative management and adverse outcomes following blunt splenic injury [21].

Another key finding of this study is that even patients with spleen AIS 5 injuries were successfully managed without any intervention. Specifically, 85.3% of 804 patients managed nonoperatively survived, a rate that rivals that of surgery at 84.9% of 2106 patients but is less than the 92.6% survival of 108 SAE patients. Indeed, the linear correlation matrix identified the global injury burden, and not splenic injury severity or management thereof, as strongly associated with mortality. Further, while cause of death is not specified in the PUF files, it can be stated that in the 8703 patients without severe injuries (is AIS≤2) in the non-abdominal body regions, amongst the 227 mortalities, 65 had an abdomen AIS higher than the spleen injury severity, with 4 patients having a principally nonsurvivable abdomen AIS of 6 and 16 having an abdomen AIS of 5. Hence, in these 20 the non-splenic abdominal injuries would have been associated with mortality.

In support of the above findings, a recent multi-center trial of hemodynamically stable patients with spleen AIS3 (with large hemoperitoneum)/4/5 (with persistent splenic vascularization) injuries demonstrated that prophylactic SAE as compared to nonoperative management had a comparable percentage of patients who had over 50% viable spleen on CT at 1 month, no difference in complication rate by day 5, or between days 5–30, with fewer splenic artery pseudoaneurysms (1.5% vs 29.2%) and less secondary SAEs (1.5% SAE vs 29.2% nonoperative) [22]. Given the comparable outcomes in terms of splenic salvage and complications, these authors opined that both routine SAE for higher severity splenic injuries or routine CT surveillance at approximately post injury day 5, with intervention as needed, were acceptable. Our data suggest however that during the study timeframe, this is not what was occurring (S1 Fig), with fewer higher-grade injuries managed with observation.

The feasibility of initial nonoperative management was also noted in a propensity matching study of the TQIP database from 2013–2014 for 2,746 patients with grade IV and grade V blunt splenic injury where 52.2% were attempted to be treated non-operatively [3]. The study noted a nonoperative failure rate of 20%, but lower in-hospital mortality in the attempted nonoperative management group. Findings from TQIP studies in higher grade BSI in adult patients argue against other studies which demonstrate increased mortality with delay to laparotomy [3, 16, 23]. In this study, only 921/9209 (10.4%) surgical patients underwent splenic surgery after 24 hours, of which 104 (1.1%) had spleen AIS5; arguably these could be considered as failures of nonoperative management.

Finally, a study examining the time to failure of nonoperative management found 0% of spleen AIS grade V failures occurred after 24 hours, in contrast to the 1% in this study, again lending support to the notion of hemodynamic stability being the prime determinant of management strategy [24]. Univariate data from this study extends this concept by demonstrating that except at extremely low initial ED SBP, surgery has higher mortality than observational management. SAE has comparable to lower unadjusted mortality than surgery. The corresponding multivariate model demonstrates that SAE is superior to surgery, especially at normotension.

### Limitations

Among the limitations of this study is that it is retrospective review of an administrative database and is subject to selection bias. It is not possible to determine why patients were treated with one strategy as opposed to another. Further, as 11996 patients did not have a mechanism specified, the separate regression analysis with mechanism of injury has decreased utility. Additionally, the number of patients receiving SAE with splenic surgery, is comparatively low, and time to SAE was not able to be determined, and hence these data not amenable to further analysis. The data considers the single recorded index ED SBP and not subsequent interventions that may affect the SBP and therefore guide management. Transfusion of blood/blood products within 4 hours was included in the models to help in part mitigate this issue. Albeit, it is not possible to determine if the lower rate of blood and blood product transfusions in the observational management group is secondary to their lower overall splenic injury severity or from selection bias or both. Additionally, this study covers data from 2013 to 2016, which is before the 2018 AAST splenic injury severity update. While it may be argued that the data is older and does not account for the updated scale of splenic injury severity, we chose this period as the AIS coding manual used until recent does not incorporate the updated splenic injury severity definitions. Therefore, analysis of data from PUF files after 2018 would have been more confusing. The possibility that some of the observed mortality benefit for SAE vs splenic surgery at lower splenic AIS in the entire patient cohort may have been because splenic artery pseudoaneurysms or arteriovenous fistulas would have been assigned higher spleen AIS of 4/5 if they had been graded using the update is acknowledged and warrants further study. Finally, time of splenic artery angioembolization was not consistently identifiable, nor was the method of SAE able to be delineated, i.e. proximal SAE (proximal to Great Pancreatic artery), distal splenic artery embolization (distal to Great Pancreatic Artery), selective embolization of the bleeding splenic artery branch (i.e. distal SAE), or non-selective embolization of entire spleen using gelfoam/polyvinyl alcohol particles etc [25]. Complications such as splenic abscess and infarcts are less with proximal SAE compared to all other groups. With increasing awareness about proximal SAE, the complication rate associated with embolization may decrease with time.

## Conclusions

Data from the TQP database, after adjustment for multiple confounders using advanced modeling techniques, demonstrated that splenic injury severity is not the prime determinant of in-hospital mortality or complications following nonpenetrating trauma in adults. Indeed, even grade 5 splenic injuries were successfully managed without any intervention, with comparable mortality rates as in the intervention arms. In the setting of severe injuries outside of the abdomen, SAE had lower mortality than surgery and observational management at spleen AIS 3/4/5. When considering index ED SBP, SAE again had lower mortality than surgery for almost all non-zero SBP. The debate will continue as we develop new techniques, but the detailed statistical analysis techniques support SAE in these high-risk settings. Future TQP studies incorporating the revised AIS splenic injury classification scheme may yield additional insights into optimal patient selection.

## Supporting information

S1 Fig A graphical representation incorporating ISS range with splenic injury severity, management strategy, and number of patients. The colors indicate splenic injury severity (spleen AIS 2—red; spleen AIS 3—blue; spleen AIS 4—green; spleen AIS 5—purple. The x-axis indicates the management group (group 1—observation, group 2—SAE, group 3—splenic surgery, group 4—SAE + splenic surgery). The y-axis indicates the number of patients. The z-axis indicates the range of ISS. Group 1 has many patients with low severity spleen injury (AIS2). Group 2 has more patients with intermediate severity spleen injury (ie spleen AIS 3 or 4). Groups 3 and 4 have more patients with higher grade splenic injury (AIS4/AIS5) in the higher ISS range groups.
(TIF)

S2 Fig Linear correlation plot of potential risk factors, complications, and mortality. Stronger positive correlations have darker shades of blue with bigger circles, while stronger inverse correlations have darker shades of orange with bigger circles. Splenic injury severity (spleen AIS) has little correlation with complications and mortality. Overall injury burden (ISS) has greater association with complications and mortality.
(TIF)
