# Supplementary figures and images for: Are interventional radiology techniques ideal for nonpenetrating splenic injury management: Robust statistical analysis of the Trauma Quality Program database
Source: PLoS One. 2024 Dec 31;19(12):e0315544. doi: 10.1371/journal.pone.0315544 (PMC11687693; doi:10.1371/journal.pone.0315544)

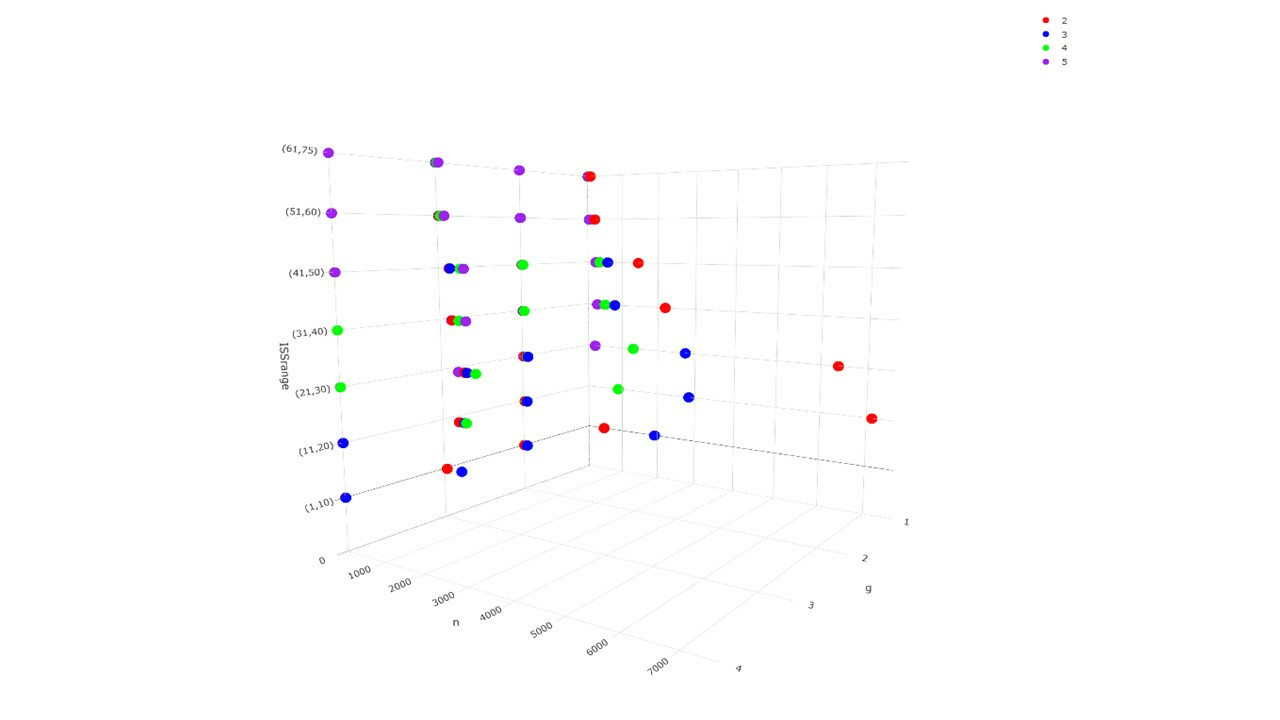

Supplement: S1 Fig — The colors indicate splenic injury severity (spleen AIS 2—red; spleen AIS 3—blue; spleen AIS 4—green; spleen AIS 5—purple. The x-axis indicates the management group (group 1—observation, group 2—SAE, group 3—splenic surgery, group 4—SAE + splenic surgery). The y-axis indicates the number of patients. The z-axis indicates the range of ISS. Group 1 has many patients with low severity spleen injury (AIS2). Group 2 has more patients with intermediate severity spleen injury (ie spleen AIS 3 or 4). Groups 3 and 4 have more patients with higher grade splenic injury (AIS4/AIS5) in the higher ISS range groups. (TIF) [file pone.0315544.s001.tif]

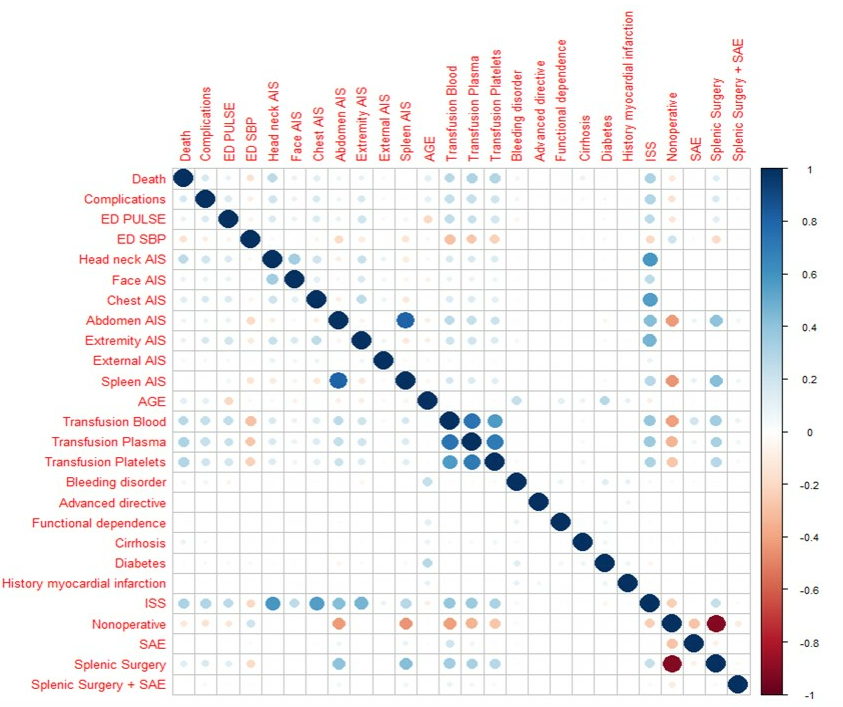

Supplement: S2 Fig — Stronger positive correlations have darker shades of blue with bigger circles, while stronger inverse correlations have darker shades of orange with bigger circles. Splenic injury severity (spleen AIS) has little correlation with complications and mortality. Overall injury burden (ISS) has greater association with complications and mortality. (TIF) [file pone.0315544.s002.tif]
